# Primary Progressive Aphasia as a Prodromal State of Dementia With Lewy Bodies: A Case Report

**DOI:** 10.3389/fneur.2020.00049

**Published:** 2020-02-18

**Authors:** Hiroyuki Watanabe, Manabu Ikeda, Etsuro Mori

**Affiliations:** ^1^Department of Behavioral Neurology and Neuropsychiatry, United Graduate School of Child Development, Osaka University, Suita, Japan; ^2^Department of Psychiatry, Graduate School of Medicine, Osaka University, Suita, Japan; ^3^Brain Function Center, Nippon Life Hospital, Osaka, Japan

**Keywords:** primary progressive aphasia, dementia with Lewy bodies, visual hallucinations, cholinesterase inhibitor, donepezil

## Abstract

Dementia with Lewy bodies (DLB) is the second most common form of dementia in the elderly, and various clinical symptoms, including olfactory dysfunction, dysautonomia, depression, and rapid eye movement sleep behavior disorders (RBD), occur in patients with the prodromal state of DLB. We herein describe a case of a 72-years-old right-handed woman who exhibited primary progressive aphasia (PPA) as a prodromal state of DLB and took cholinesterase inhibitors (donepezil). At 4.5 years after aphasia onset, she exhibited all the core clinical features of DLB, including visual hallucinations, fluctuating cognition, RBD, and Parkinsonism, as well as progressive language impairment. She showed reduced dopamine transporter (DAT) uptake (assessed by DAT single-photon emission computed tomography imaging) in the striatum and decreased cardiac uptake (determined by ^123^I-metaiodobenzylguanidine myocardial scintigraphy), which are indicative biomarkers of DLB. Thus, this patient met all the criteria for probable DLB. Notably, the unique feature of this case was the presentation of PPA, which is seldom observed in typical DLB. Moreover, cholinergic enhancement (donepezil, 5 mg daily) improved her language function and global cognitive function, although mild aphasia remained. The findings provide valuable insights into the spectrum of the prodromal state of DLB and shed light on the development of the medication for PPA caused by cholinergic insufficiency.

## Introduction

Primary progressive aphasia (PPA) is a neurological syndrome that presents with language impairment as the most salient feature. The most widely applied criteria were proposed for three clinical syndromic variants of PPA: non-fluent/agrammatic (nfvPPA), semantic (svPPA), and logopenic (lvPPA) (1). Several pathologies have been demonstrated in PPA. The pathology of nfvPPA is characterized by tauopathies, such as corticobasal degeneration, progressive supranuclear palsy, or frontotemporal lobar degeneration-tau (1, 2). The pathology of svPPA most often includes frontotemporal lobar degeneration-TDP-43 (1, 2). The pathology of lvPPA is usually Alzheimer's disease (AD) (1, 2). However, Lewy body disease (LBD), including dementia with Lewy bodies (DLB), is rarely reported in patients with PPA.

DLB is the second most common type of dementia, after AD, in the elderly (3). The core clinical features of DLB include visual hallucinations, fluctuating cognition, rapid eye movement sleep behavior disorders (RBD), and motor symptoms of parkinsonism, as well as cognitive impairment characterized by deficits of attention, executive function, and visual perception (4). Various clinical symptoms, including olfactory dysfunction, dysautonomia, depression, and RBD, are observed in patients with the prodromal state of DLB (before or at the onset of memory loss) (5–8). Moreover, recent evidence suggests the occurrence of PPA in patients with prodromal DLB (9–11). We herein present a case of a 72-years-old woman who had PPA as a prodromal state of DLB and took cholinesterase inhibitors (donepezil). The findings would further expand our knowledge on the spectrum of prodromal DLB and on the therapeutic effects of cholinesterase inhibitors on PPA caused by cholinergic insufficiency.

## Case Description

**Figure 1:**
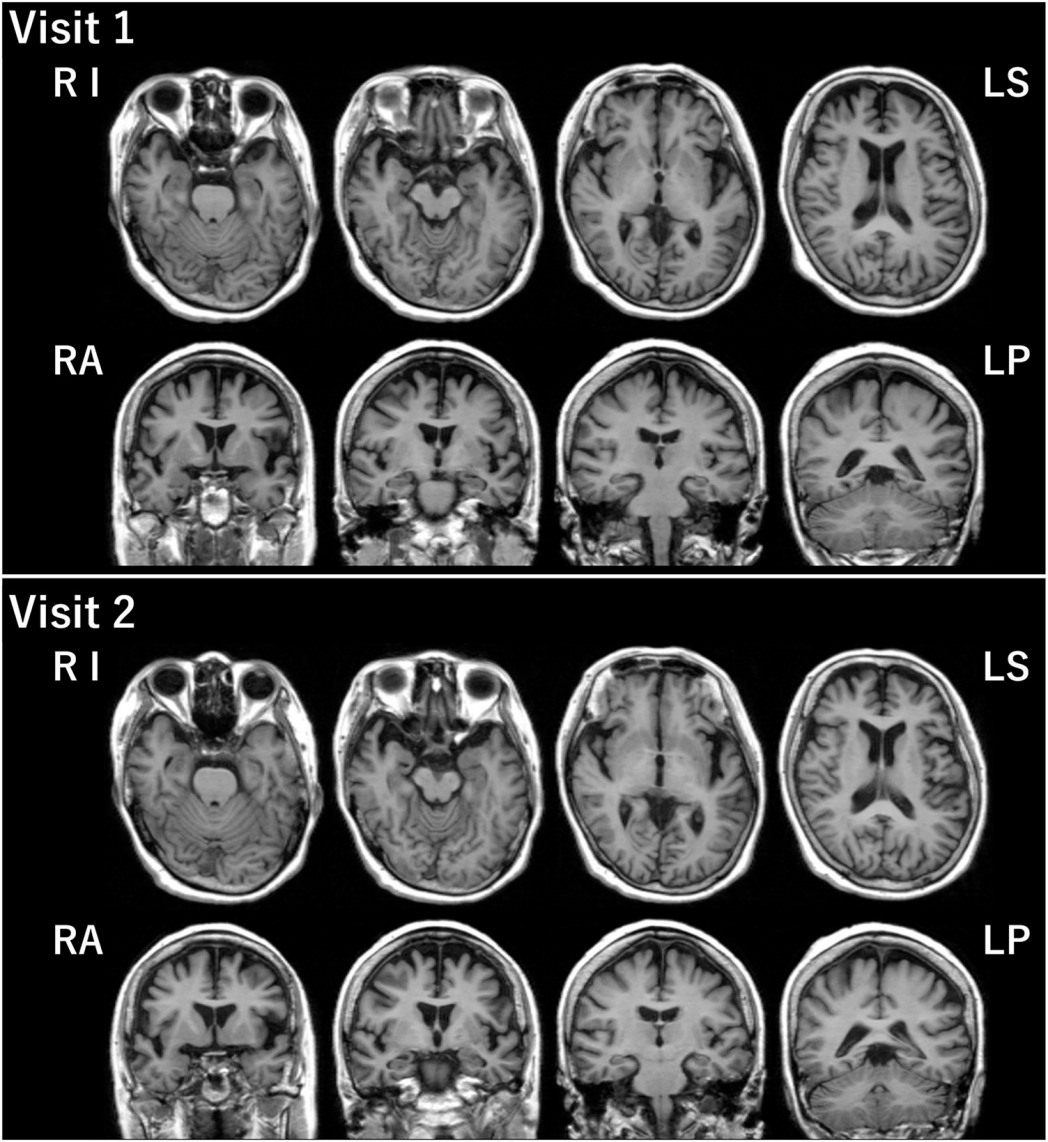
Brain MRI. Brain MRI at visits 1 and 2 revealed relative preservation of the medial temporal lobe and left-sided predominant mild atrophy in the bilateral perisylvian area. LP, left posterior; LS, left superior; RA, right anterior; RI, right inferior.

**Figure 2:**
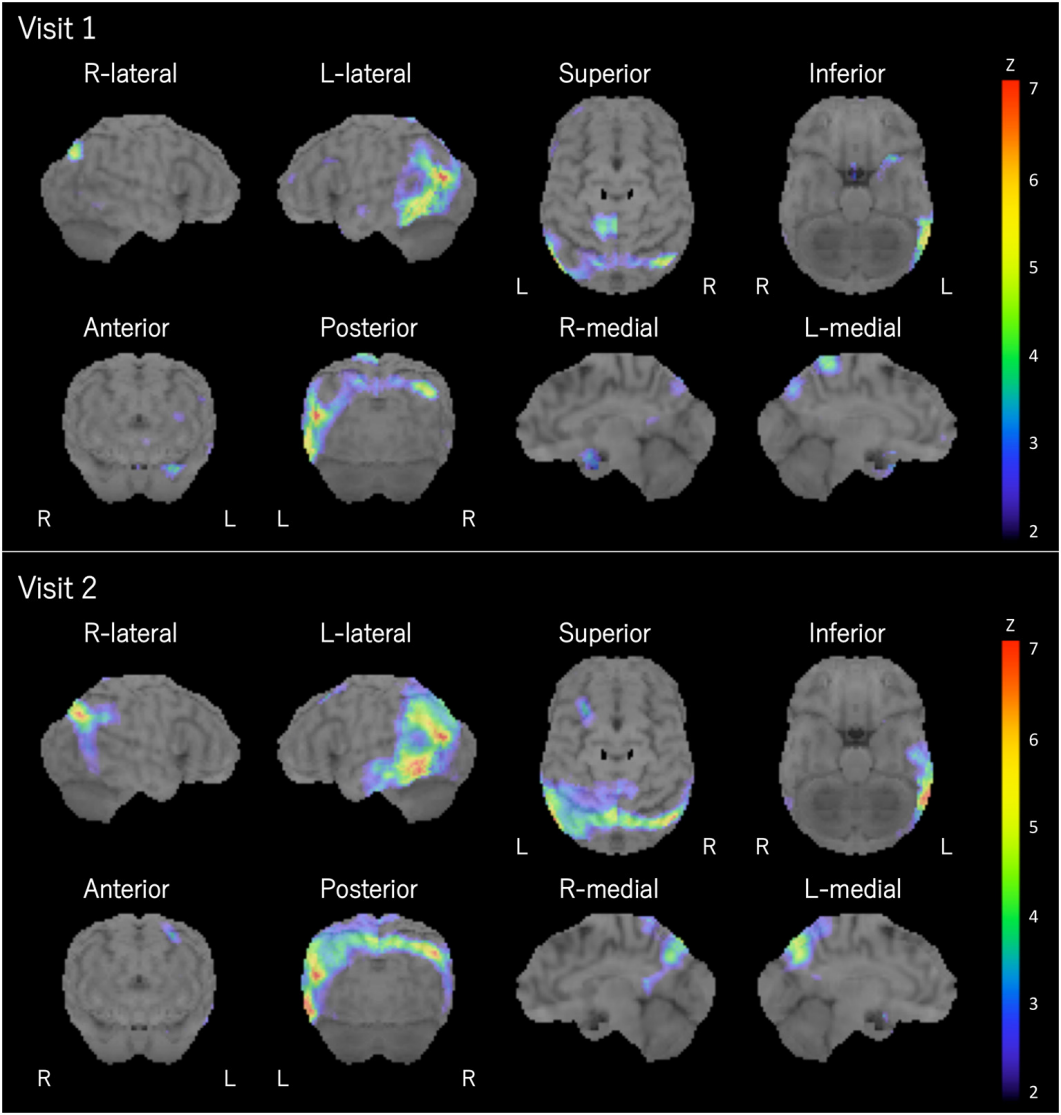
Brain SPECT. Brain SPECT analyzed with an easy *Z*-score imaging system at visits 1 and 2 revealed predominant left-sided hypoperfusion in the temporoparietal lobe. L, left; R, right.

The patient was a right-handed woman with 14 years of education. At visit 1, she was 71 years old, and she visited our hospital because of gradually progressive difficulty in thinking of words and speaking. Thinking of words and speaking had concurrently become challenging at the age of around 67 years. She was diagnosed with depression when she was 68 years old, and she recovered from depression after undergoing the recommended treatment for 3 months. Except for depression, her medical history was unremarkable. At the initial exam, physical and neurological examinations and routine laboratory tests showed no abnormalities. Brain magnetic resonance imaging (MRI) revealed the relative preservation of the medial temporal lobe and left-sided predominant mild atrophy in the bilateral perisylvian area (Figure 1). There was no evidence of hemorrhage or ischemic lesions. N-iso-propyl-p-[^123^I] iodoamphetamine single-photon emission computed tomography (SPECT) data analyzed with an easy *Z*-score imaging system found predominant left-sided hypoperfusion in the temporoparietal lobe (Figure 2).

At visit 2 (4.5 years post-symptom onset), her language impairments had progressed. The pattern of her atrophy on MRI did not progress at visit 2 compared with visit 1 (Figure 1). The patterns of hypoperfusion on SPECT at visit 2 were similar to those at visit 1. However, hypoperfusion at visit 2 was expanded mainly in the left temporoparietal lobe and progressed compared with that at visit 1 (Figure 2). In addition, she started experiencing recurrent visual hallucinations at night (other people standing in her bedroom), fluctuations of cognition, and RBD, which are characteristic symptoms of DLB (4). Moreover, neurological examinations showed mild bilateral hand rigidity. Dopamine transporter (DAT) SPECT imaging with ^123^I-FP-CIT found reduced DAT uptake in the striatum [Specific Binding Ratio (SBR) = R: 4.46, L: 4.08, Avg: 4.27]; the finding is in accordance with the notion that parkinsonism is a characteristic symptom of DLB (4). Her myocardial accumulation of ^123^I-metaiodobenzylguanidine (^123^I-MIBG) decreased (H/M = early: 2.07, delayed: 1.52). She did not have cardiac disease, and she was not being treated with drugs that might affect DAT scan or ^123^I-MIBG test. Therefore, LBD was suspected at visit 2. After neuropsychological and behavioral examinations, she was prescribed donepezil.

At visit 3 (5 years post-symptom onset), she underwent detailed neuropsychological and neuropsychiatric assessments to investigate the impact of cholinergic enhancement on cognitive and behavioral impairments. In this study, visits 1 and 2 were baseline sessions without medication, and visit 3 was the follow-up session with medication (donepezil). The follow-up sessions started 3 months later after the dosage of donepezil was increased to 5 mg/day. Written informed consent was obtained from the patient and her family members for the participation of this study and publication of this case report and any accompanying images.

## Clinical Assessments

### Neuropsychological Assessments

Neuropsychological assessments were performed by an experienced speech and language therapist (HW). The patient underwent the Wechsler Adult Intelligence Scale–Third Edition (WAIS-III) and the Wechsler Memory Scale-Revised (WMS-R) at visits 1 and 3, the Addenbrooke's Cognitive Examination-Revised (ACE-R) (12, 13) at visits 2 and 3, and the Digit Span and the Spatial Span tests at each visit. She did not undergo WAIS-III and WMS-R at visit 2 because there were only 6 months since visit 1. ACE-R was used to assess general intelligence and memory at visit 2.

### Language Assessments

Bedside language assessments (14) were performed by an experienced behavioral neurologist (EM) and a speech and language therapist (HW). In addition, in-depth evaluations of global language ability were performed by HW using the Western Aphasia Battery (WAB) (15, 16), and the Token test was performed by HW to determine long and syntactic comprehension at each visit.

### Behavioral and Neuropsychiatric Assessments

Behavioral and neuropsychiatric assessments were performed by an experienced behavioral neurologist (EM) and a speech and language therapist (HW). The patient's caregivers underwent the Neuropsychiatric Inventory 12-item version (NPI-12) (17) to assess behavioral and neuropsychiatric symptoms and the Cognitive Fluctuation Inventory (18) to determine fluctuation in cognition at each visit. For the delusion domain, we separately assessed persecutory delusions and delusional misidentifications (19). Moreover, she underwent the noise pareidolia test, in which visual hallucination-like illusions were evoked and measured (19, 20). We used illusions (pareidolia) as a surrogate marker of visual hallucinations (19, 20) at visits 2 and 3.

## Results

### Neuropsychological Assessments

The results of standard neuropsychological assessments are presented in Table 1. Overall, her scores on neuropsychological assessments, except for language assessment, across visits 1–3 were normal. Digit and spatial span performances were normal at each visit, suggesting preserved verbal and visual short-term memory. At visit 1, the patient's intelligence (WAIS-III) and memory (WMS-R) scores were normal.

**Table 1:** Demographic information and performances on neuropsychological and neuropsychiatric tests at each visit.

	**Visit 1 (baseline)**	**Visit 2 (baseline)**	**Visit 3 (follow-up)**	**Normative data [mean (SD)]**
Age (years)	71	71.5	72	
Disease duration (years since symptom onset)	4	4.5	5	
**WAIS-III**				
Full IQ	100	DNT	104	100.0 (15.0)
Verbal IQ	104	DNT	109	100.0 (15.0)
Performance IQ	95	DNT	98	100.0 (15.0)
**WMS-R**				
General memory index	93	DNT	109	100.0 (15.0)
Verbal memory index	87	DNT	101	100.0 (15.0)
Visual memory index	109	DNT	123	100.0 (15.0)
Attention/concentration index	86	DNT	98	100.0 (15.0)
Delayed recall index	99	DNT	118	100.0 (15.0)
**ACE-R**				
Total [100]	DNT	86	96	91.1 (8.4)
Attention/orientation [18]	DNT	18	18	17.5 (1.2)
Memory [26]	DNT	21	25	21.0 (4.8)
Verbal fluency [14]	DNT	8	12	12.3 (2.0)
Language [26]	DNT	23	25	24.3 (1.7)
Visuospatial [16]	DNT	16	16	15.9 (0.2)
**Digit span**	F7B4	F6B4	F6B5	
**Spatial span**	F5B4	F5B4	F5B4	
**NPI**				
Persecutory delusions	0	0	0	
Delusional misidentifications	0	0	0	
Hallucinations	0	1	1	
Agitation/aggression	0	0	0	
Depression	0	3	2	
Anxiety	0	0	2	
Euphoria	0	0	0	
Apathy	0	4	3	
Disinhibition	0	0	0	
Irritability/lability	0	0	0	
Aberrant motor behavior	0	0	0	
Sleep disturbances	0	2	2	
Eating abnormalities	0	8	8	
Fluctuations in cognition	0	2	2	
**Noise pareidolia tests**				
Pareidolias [32]	DNT	3	0	0.2 (0.4)
Detection misses [8]	DNT	2	2	0.0 (0.0)

*The maximum score is noted in each row header; ACE-R, Addenbrooke's Cognitive Examination-Revised; B, backward; DNT, did not test; F, forward; IQ, intelligence quotient; NPI, Neuropsychiatric Inventory; WAIS-III, Wechsler Adult Intelligence Scale-Third Edition; WMS-R, Wechsler Memory Scale-Revised*.

At visit 2, she showed low average scores on the verbal fluency and language subcomponent of ACE-R because of her language impairments. As a result, the total score of ACE-R was lower than the average score. However, her attention/orientation, memory, and visuospatial subcomponent scores on ACE-R were ≥the average scores. These findings indicated that her cognitive functions, except for language ability, were normal at visit 2.

Improvements were observed in all neuropsychological assessments at visit 3 compared with visits 1 and 2, although she showed no other obvious cognitive impairments, except for aphasia, at visits 1 and 2. Improvements were also observed in full-scale intelligence quotient (IQ), verbal IQ, and performance IQ of the WAIS-III and general memory index, verbal memory index, visual memory index, delayed recall index, and attention/concentration index of the WMS-R at visit 3 compared with visit 1. Moreover, improvements were observed in total scores of the ACE-R and memory, verbal fluency, and language subcomponent scores of the ACE-R. However, improvements were not observed in attention/orientation and visuospatial subcomponent scores on the ACE-R because of the ceiling effect. Therefore, these results revealed that donepezil improved her cognitive functions, including language functions.

### Language Assessments

At visit 1, she retained awareness of her language impairments, and her motivation to communicate was well-preserved, as demonstrated by bedside language assessments. Her spontaneous speech was characterized by a slow rate with frequent pauses due to word-finding difficulty. However, her grammar, syntax, average phrase length, and prosody were normal. She had no apraxia of speech or agrammatism (a core feature of nfvPPA) or paraphasia. She had no single-word comprehension deficit or loss of word meaning (a core feature of svPPA). Therefore, anomia was the predominant feature of her language impairments. The results of standard language assessments are presented in Table 2. At visit 1, she had mild aphasia, as indicated by a WAB-Aphasia quotient (AQ) score of 90.6. She might have lvPPA because of her spontaneous speech characterized by word-finding difficulty and lower-than-average WAB-Repetition score (16). However, her sentence repetition ability was mostly preserved [9.0 in our patient; mean scores of 32 healthy individuals, 22 anomic aphasic patients, and 7 conduction aphasic patients: 9.9 ± 0.3, 9.0 ± 1.0, and 5.4 ± 3.5, respectively (16)], and she had intact verbal short-term memory (Digit-span performance: forward, 7; backward, 4). Moreover, she had no phonemic paraphasias (a characteristic symptom of lvPPA). Therefore, she met core criteria for PPA, but she did not meet classification for any variants of PPA (1). The pattern of her aphasia was similar to progressive fluent aphasia (21–23), anomic subtype of PPA (24–27), or that observed in patients with anomic aphasia following stroke because of preserved auditory comprehension (WAB-Auditory comprehension score, 10; Token test score, 166).

**Table 2:** Performances on language tests at each visit.

	**Visit 1 (baseline)**	**Visit 2 (baseline)**	**Visit 3 (follow-up)**	**Normative data [mean (SD)]**
Types of aphasia	Anomic	Anomic	Anomic	
**WAB**				
Aphasia quotient [100]	90.6	87.2	92.2	97.7 (3.0)
Fluency [10]	9	9	9	10.0 (0)
Information content [10]	8	8	9	9.7 (0.6)
Auditory comprehension [10]	10	8.5	9.3	9.8 (0.1)
Repetition [10]	9	9.6	9.6	9.9 (0.3)
Naming [10]	9.3	8.5	9.2	9.5 (0.6)
Reading [10]	9.3	9.1	9.7	9.5 (0.8)
Writing [10]	10	9.6	9.8	9.6 (1.0)
Praxis [60]	60	60	60	59.8 (0.7)
Calculation [24]	24	24	24	23.1 (2.3)
**Token test** [166]	166	155	157	163.6 (2.0)

*The maximum score is noted in each row header; WAB, Western Aphasia Battery*.

At visit 2, her main complaint was gradually progressive difficulty in speaking. Particularly, a slow rate in her spontaneous speech with frequent pauses due to word-finding difficulty deteriorated in bedside language assessments at visit 2 compared with visit 1. Moreover, there was a considerable degree of circumstantiality in her spontaneous speech, and the content tended to be empty. However, she could produce long sentences with correct grammatical structures. She had no dysprosody, apraxia of speech, agrammatism, paraphasia, single-word comprehension deficit, or loss of word meaning. As observed at visit 1, anomia was the predominant feature of her language impairments at visit 2. As demonstrated by bedside language assessments at visit 2, her aphasia had progressed, as indicated by a WAB-AQ score of 87.2. Particularly, the decline of word-finding (WAB-naming score, 8.5) and auditory comprehension ability (WAB-Auditory comprehension scores, 8.5; Token test score, 155) was observed. There were mild fluctuations in the WAB-Repetition test between visits 1 and 2; however, overall, she preserved mostly intact repetition ability. The pattern of her aphasia did not change from that of anomic aphasia.

At visit 3, the content in her spontaneous speech increased and improved in bedside language assessments. However, circumstantiality and a slow rate with frequent pauses in her spontaneous speech due to word-finding difficulty remained. She had no dysprosody, apraxia of speech, agrammatism, paraphasia, single-word comprehension deficit, or loss of word meaning. Therefore, as observed at visits 1 and 2, anomia was also the predominant feature of her language impairments at visit 3. As demonstrated by bedside language assessments at visit 3, her language functions at visit 3 had improved from those at visits 1 and 2, as indicated by a WAB-AQ score of 92.2. In particular, her word-finding difficulty had improved in spontaneous speech, but her mild anomic aphasia remained. In addition, improvements were observed in AQ, Information content, Repetition, and Reading of the WAB at visit 3 compared with visit 1. Moreover, improvements were also observed in AQ, Information content, Auditory comprehension, Naming, Reading, and Writing of the WAB, as well as the Token test, at visit 3 compared with visit 2. Therefore, these results revealed the effectiveness of donepezil in her language improvements.

### Behavioral and Neuropsychiatric Assessments

Results of behavioral and neuropsychiatric assessments are presented in Table 1. At visit 1, she showed no behavioral and psychiatric symptoms.

At visit 2, she had increased neuropsychiatric symptoms, including depression, apathy, and eating abnormalities and enhanced characteristic symptoms of DLB, including hallucinations, fluctuations in cognition, and sleep disturbances on the NPI. She showed pareidolic responses (visual hallucination-like illusions) in the noise pareidolia test.

At visit 3, although improvements were observed in depression and apathy, she showed increased anxiety on the NPI. No improvement in characteristic symptoms of DLB, including hallucinations, fluctuations in cognition, and sleep disturbances, was observed on the NPI. However, she showed reduced pareidolic responses in the noise pareidolia test and improved visual hallucination-like illusions.

## Discussion

We herein present a case of PPA due to LBD. The patient exhibited all the core clinical features of DLB, including visual hallucinations, fluctuating cognition, RBD, and parkinsonism, as well as progressive cognitive decline. Moreover, she exhibited the supportive clinical features of DLB, including apathy, anxiety, and depression, as assessed with the NPI. In addition, she showed reduced DAT uptake (assessed by DAT SPECT imaging) in the striatum and decreased cardiac MIBG uptake (assessed by ^123^I-MIBG myocardial scintigraphy), which are indicative biomarkers of DLB. Thus, this patient met all the criteria for probable DLB (4). Notably, the unique feature of this case was the presentation of aphasia, which is seldom observed in typical DLB.

To date, two patients (28, 29) with nfvPPA, two patients (9, 11) with lvPPA, and eight patients (10) with PPA have been reported in the literature. Based on the detailed neuropsychological and language assessments, the patient in this case report met the core criteria for PPA (1) but did not meet classification for any variants of PPA (1). Several studies have demonstrated the existence of an additional, atypical variant of PPA (26, 30, 31), indicating that the established consensus criteria may not account for the full range of clinical syndromic variants of PPA. The pattern of her aphasia and the imaging findings (predominant left-sided atrophy and hypoperfusion of the temporoparietal lobe) were similar to those observed in patients with anomic aphasia (32, 33). Similar patients in other studies have been demonstrated to have progressive fluent aphasia (21–23) or anomic subtype of PPA (24–27). Therefore, the findings in the present case report may help improve the PPA criteria (1), and LBD should be considered one of the pathologies of PPA.

Various clinical symptoms, including olfactory dysfunction, dysautonomia, depression, and RBD, occur in patients with the prodromal state of DLB (before or at the onset of memory loss) (5–8). Moreover, recent evidence suggests the existence of prodromal DLB characterized by PPA (9–11). After 4.5 years (post-language impairment onset), the patient in this study developed visual hallucinations, fluctuations in cognition, parkinsonism, and RBD, and she thus fulfilled the criteria of probable DLB (4). Therefore, the present findings suggest the existence of prodromal DLB characterized by PPA and provide insights into the spectrum of the prodromal state of DLB.

The effectiveness of cholinesterase inhibitors, such as donepezil and galantamine, in cognition, global function, and visual hallucinations in DLB has been demonstrated in several studies (4, 34–36), and the effectiveness of donepezil in post-stroke aphasia has been established in a randomized placebo-controlled study (37). In addition, the effectiveness of galantamine in PPA patients with unspecified subtypes was investigated in 18-weeks open-label and 8-weeks randomized placebo-controlled studies, and language function remained stable in some of the galantamine-treated PPA patients compared with placebo-treated patients (38–40). However, the efficacy of donepezil in PPA has not been investigated in a randomized clinical trial. In particular, because the most common pathology underlying lvPPA is AD pathology, aphasic symptoms might be improved with cholinesterase inhibitors (2, 39). Our patient had not received speech and language therapy for aphasia. Notably, after administering donepezil at visit 3, improvements were observed in language and global cognitive functions, but not in visual hallucinations, although one of the well-known mechanisms of visual hallucinations in DLB is cholinergic insufficiency. In addition, she showed improvements in the number of pareidolias (a surrogate marker of visual hallucinations) after administering donepezil, as observed in a previous study (19). Notably, cholinergic deficits are markedly evident in DLB compared to AD (41). In fact, these present results revealed the effectiveness of cholinesterase inhibitors in her language and global cognitive functions. Therefore, the current findings shed light on the development of medication for PPA caused by cholinergic insufficiency.

The present study has several limitations. First, our patient did not undergo additional tests, such as the Unified Parkinson's Disease Rating Scale (UPDRS) part III, to assess motor symptoms or further formal evaluation, such as the polysomnography, to assess RBD. Second, we used WAB as the main language test in this study. Currently, the Western Aphasia Battery revised (WAB-R) (42) developed for assessing aphasia following stroke has been widely used in assessing PPA (43). However, a recent study demonstrated that the WAB-R AQ underestimated the presence of PPA, and when the WAB-R AQ was used to detect aphasia in neurological disorders, the WAB-R connectionist classification of aphasia following stroke did not distinguish various variants of PPA because aphasia was frequently classified as anomic aphasia in PPA (22). Therefore, we might make a wrong diagnosis of anomic aphasia, although we excluded all three types of PPA based on not only the results of WAB but also qualitative assessments performed by an experienced behavioral neurologist (EM) and a speech and language therapist (HW). In addition, because the WAB alone is insufficient to detect or fully characterize PPA, other neuropsychological tests should be considered to identify the key symptoms that can be used to discriminate among PPA variants (14, 22, 43). Third, improvements in language and global cognitive functions after administering donepezil at visit 3 might reflect fluctuating cognition of DLB, although her attention/concentration index of the WMS-R and attention/orientation subcomponent scores on ACE-R were normal at each visit. Fourth, we did not analyze the cerebrospinal fluid biomarkers (tau, beta-amyloid, and alpha-synuclein), and no pathological examinations were conducted in this patient. Therefore, further investigations are needed to determine pathological changes.

## Conclusions

This case report describes a case of PPA as the prodromal state of DLB. This case report revealed the effectiveness of cholinesterase inhibitors in her language and global cognitive functions. Our findings provide insights into the spectrum of the prodromal state of DLB and shed light on the development of the medication for PPA caused by cholinergic insufficiency.

## Data Availability Statement

The raw data supporting the conclusions of this article will be made available by the authors, without undue reservation, to any qualified researcher.

## Ethics Statement

Ethical review and approval was not required for the study on human participants in accordance with the local legislation and institutional requirements. The patients/participants provided their written informed consent to participate in this study.

## Informed Consent

Written informed consent was obtained from the participant for the publication of any identifiable information.

## Author Contributions

HW acquired the data, designed the study, and drafted the manuscript. MI supervised the study and helped to draft the manuscript. EM acquired the data, supervised the study, and helped to draft the manuscript.

### Conflict of Interest

The authors declare that the research was conducted in the absence of any commercial or financial relationships that could be construed as a potential conflict of interest.
